# Effects of Micro(nano)plastics on Anaerobic Digestion and Their Influencing Mechanisms

**DOI:** 10.3390/microorganisms13092118

**Published:** 2025-09-10

**Authors:** Xinghua Qi, Hezhen Wang, Yixuan Li, Jing Liu, Jiameng Sun, Wanli Zhang, Wanli Xing, Rundong Li

**Affiliations:** School of Energy and Environment, Shenyang Aerospace University, No. 37 Daoyi South Avenue, Shenyang 110136, China

**Keywords:** microplastics, nanoplastics, anaerobic digestion, organic waste

## Abstract

Micro(nano)plastics are important emerging contaminants and a current research hotspot in the environmental field. Micro(nano)plastics widely exist in various organic wastes such as waste sludge, food waste (FW) and livestock manure and often enter into digesters along with anaerobic digestion (AD) treatment of these wastes, thereby exerting extensive and profound influences on anaerobic process performance. This study reviews sources of micro(nano)plastics and their pathways entering the anaerobic system and summarizes the quantities, sizes, shapes and micromorphology of various micro(nano)plastics in waste sludge, FW, livestock manure, yard waste and municipal solid waste. The current advances on the effects of multiple micro(nano)plastics mainly polyvinyl chloride (PVC), polystyrene (PS) and polyethylene (PE) with different sizes and quantities (or concentrations) on AD of organic wastes in terms of methane production, organic acid degradation and process stability are comprehensively overviewed and mechanisms of micro(nano)plastics affecting AD involved in microbial cells, key enzymes, microbial communities and antibiotic resistance genes are analyzed. Meanwhile, coupling effects of micro(nano)plastics with some typical pollutants such as antibiotics and heavy metals on AD are also reviewed. Due to the extreme complexity of the anaerobic system, current research still lacks full understanding concerning composite influences of different types, sizes and concentrations of micro(nano)plastics on AD under various operating modes. Future research should focus on elucidating mechanisms of micro(nano)plastics affecting organic metabolic pathways and the expression of specific functional genes of microorganisms, exploring the fate and transformation of micro(nano)plastics along waste streams including but not limited to AD, investigating the interaction between micro(nano)plastics and other emerging contaminants (such as perfluorooctanoic acid and perfluorooctane sulphonate) and their coupling effects on anaerobic systems, and developing accurate detection and quantification methods for micro(nano)plastics and technologies for eliminating the negative impacts of micro(nano)plastics on AD.

## 1. Introduction

Plastics are artificial chemicals with light weight, good durability and low cost. From daily life products (packaging, films, covers, bags and containers) to industrial products, medical supplies and aeronautical materials, plastics cover a wide range of fields, from short-lived, fast-moving consumer goods to long-lasting items [1,2]. In 2022, global plastic production reached 400.3 million tons and continued to rise annually [3]. However, only approximately 9% of plastic waste is recycled [4]. The production and use of plastics inevitably result in the release of a significant amount of micro(nano)plastics into the environment [5]. Microplastics generally refer to plastic particles <5 mm in size [6]. Microplastics can be divided into primary microplastics and secondary microplastics based on their sources. Primary microplastics are plastic particles smaller than 5 mm in size in the manufacturing process that are usually discharged directly into the environment in the form of small particles [7]. Secondary microplastics are small plastic particles formed from physical, chemical or biological degradation of large plastic products in the environment [7]. Micro(nano)plastics widely distributed in various environmental media such as soil, oceans, atmosphere and lakes [8,9,10]. As an emerging contaminant, micro(nano)plastics may pose some potential threats to both the environment and human health [11]. Current studies reported that micro(nano)plastics widely existed in various organic wastes such as waste sludge, food waste (FW) and livestock manure [12,13]. Li et al. analyzed seventy-nine sludge samples from twenty-eight wastewater treatment plants (WWTPs) in China and found that microplastic concentrations ranged from 1.60 × 10^3^ to 56.4 × 10^3^ particles/kg of dry sludge [14]. Similarly, Liu et al. reported that microplastic concentrations in sludge of WWTPs in Japan ranged from 81 ± 48 to 6470 ± 1490 particles/kg of dry sludge [15]. Magni et al. detected a good deal of microplastics (113 ± 57 particles/g of dry weight sludge) in activated sludge of a sewage treatment plant in Italy [16]. An investigation concerning microplastics in the grocery waste of the USA found 3.0 × 10^5^ plastic particles per kilogram of dry material [12].Yang et al. also reported an average microplastics quantity of 1250 ± 640 particles/kg in pig manure [17]. Generally, the particle densities of microplastics range from 0.88 to 1.40 g/cm^3^, lower than that of natural sediments (1.5–3.0 g/cm^3^) [18]. Micro(nano)plastics commonly found in the environment are mainly composed of polymers such as polyethylene (PE), polypropylene (PP), polystyrene (PS) and polyvinyl chloride (PVC) [19]. These polymers are synthesized through the polymerization of their corresponding monomers, and their chemical structures are also directly inherited from these monomer units. For example, PE is synthesized by polymerizing ethylene, with the chemical formula of (C_2_H_4_)n; PP is derived from the propylene monomer, with the chemical formula of (C_3_H_6_)n; PVC is formed by the polymerization of vinyl chloride, with the structure of (C_2_H_3_Cl)n; PS is prepared by styrene, with the chemical formula of (C_8_H_8_)n.

Anaerobic digestion (AD) is a sustainable waste-to-energy technology that could convert organic waste to biogas (mainly methane and carbon dioxide) by anaerobic microorganisms. Currently, AD is the predominant treatment technology of biodegradable organic wastes such as sludge, FW and livestock manure [20]. These organic wastes often contain plenty of micro(nano)plastics and they enter the AD system by the feeding of digesters together. Thus, these organic wastes are the main sources and pathways of micro(nano)plastics entering the anaerobic system [21]. Recent studies found that micro(nano)plastics often exhibit negative effects on AD performance [6]. Fu et al. investigated the impact of 54.8 nm polystyrene (PS) nanoplastics at a concentration of 0.2 g/L on hydrogen production of a pure anaerobic system of *Acetobacteroides hydrogenigenes* and found that PS nanoplastics decreased the hydrogen yield from 13.0 mL to 12.3 mL, and many PS nanoplastic particles attached to the cell membrane of *Acetobacteroides hydrogenigenes* and caused cell wall pitting [1]. Wei et al. also reported that polyvinyl chloride (PVC) microplastics with a particle size of 1 mm and concentrations of 20 to 60 particles/g total solids (TS) reduced the methane production of the AD of waste-activated sludge (WAS) to 75.8–90.6% of that of the control due to the toxic bisphenol A leached from PVC microplastics, and stronger inhibition occurred at higher concentrations [22]. Similarly, Wei et al. reported that polyethylene (PE) microplastics at concentrations of 100–200 particles/g TS reduced methane production by 12.4–27.5% of the AD of WAS [23]. This reduction might be attributed to the induction of reactive oxygen species (ROS) production by PE microplastics, which could decrease anaerobic microbial activity. In contrast, Chen et al. observed that the addition of polyamide 6 (PA6) microplastics at a dosage of 5–50 particles/g TS significantly increased the methane production of the AD of WAS from 124 ± 6 L CH_4_/kg volatile solids (VS) to 173 ± 8 L CH_4_/kg VS, representing an increase of 39.5%, and supposed that the leaching of caprolactam from PA6 enhanced the activity of key enzymes involved in acidogenesis and methanogenesis during AD [24]. Furthermore, previous studies demonstrated that micro(nano)plastics significantly enhanced abundances of antibiotic resistance genes (ARGs) in the anaerobic system, which may pose potential risks to human health and the environment [25,26,27]. Significantly, various pollutants (antibiotics, heavy metals, etc.) would be adsorbed onto micro(nano)plastics, causing negative effects on AD [5].

This study reviews mechanisms of micro(nano)plastics and their effects on AD. Firstly, sources of micro(nano)plastics and their pathways entering the anaerobic system are summarized. Then, the effects of PVC, PS, PE and other micro(nano)plastics on the AD of organic wastes are overviewed. Next, mechanisms of micro(nano)plastics affecting AD in terms of microbial cells, key enzymes, microbial community and antibiotic resistance genes are analyzed. Meanwhile, coupling effects of micro(nano)plastics with other pollutants on AD are also reviewed, and the future development direction concerning micro(nano)plastics in AD is also proposed. This study expands the understanding of the pathways and mechanisms of micro(nano)plastics affecting AD. The literature search was conducted using the database of Web of Science using “microplastics” and “anaerobic digestion” as core keywords combined by related terms such as “enzyme activity”, “microbial community” and “antibiotic resistance genes”. To ensure the comprehensiveness and scientificity of this review, all the included literature must be original and publicly published and generally focused on the effects of microplastics on anaerobic digestion.

## 2. Pathways of Micro(nano)plastics Entering the Anaerobic System and Their Characteristics

### 2.1. Sludge

**Figure 1:**
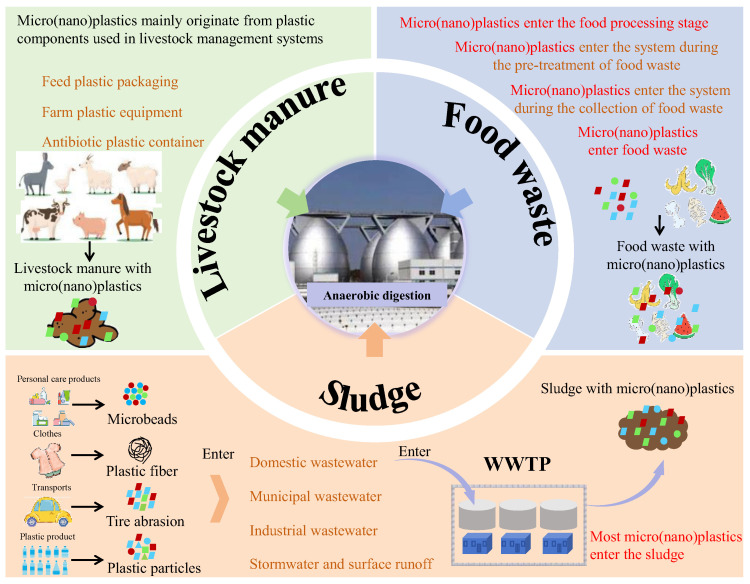
Graphical representation of micro(nano)plastics entering an anaerobic system.

**Figure 2:**
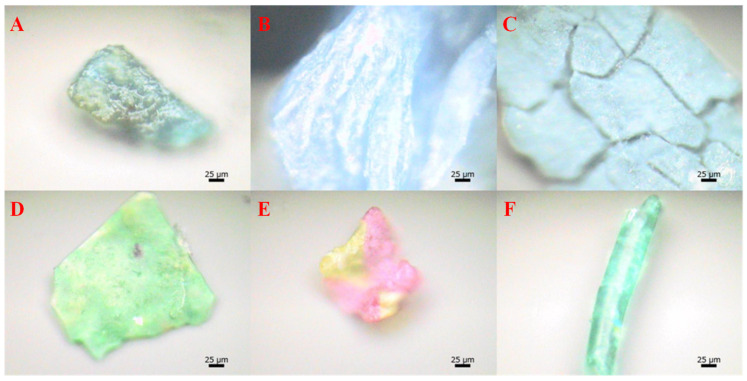
Micromorphology of microplastics in sludge [15]: (**A**) PS; (**B**) PE; (**C**) PE; (**D**) polyester; (**E**) PVC; and (**F**) PU.

Wastewater treatment plants serve as both sources and sinks of micro(nano)plastics [28] (Figure 1). Current wastewater treatment technologies do not completely eliminate micro(nano)plastics [29]. During wastewater treatment, approximately 99% of micro(nano)plastics ultimately accumulate in the sludge [30]. Figure 2 presents the microscopic morphology of microplastics in sludge samples. Table 1 summarized the characteristics of microplastics in sludge reported in previous studies. Li et al. reported that microplastics from wastewater treatment plants are mainly transferred to sludge, with average concentrations ranging from 38.6 to 104.5 particles/g dry weight of sludge [31]. Edo et al. analyzed mixed sludge and heat-dried sludge collected from Madrid, Spain, and found that microplastic concentrations reached 183 ± 84 and 165 ± 37 particles/g, respectively [11]. Zhang et al. found that microplastics abundances in sludge from four WWTPs in Guilin, China, ranged from 234.7 to 6908.3 particles/kg dry weight [5]. Similarly, microplastics abundance in activated sludge from four WWTPs in Guangzhou, China, ranged from 7 to 888 pieces/g dry weight [32]. Maw et al. reported microplastic concentrations in sludge of an WWTP in Thailand reached 20,000–228,100 particles/kg dry weight. These reports indicate that global sludge contains a large amount of microplastics [33].

The most common micro(nano)plastics found in sludge are PVC, PS, PE, polypropylene (PP), polyamide (PA) and polyethylene terephthalate (PET) [34]. These micro(nano)plastics mainly exist in the form of fragments, fibers, microbeads, films and irregular particles [14]. Li et al. reported that 63% of microplastics in sludge were fibrous, and 59.6% of fibrous microplastics were white fibers (59.6%), mainly from textile effluents and fiber manufacturing discharges [14]. Edo et al. found that polyester fibers were the main microplastics in sludge of a WWTP in Spain, followed by acrylic fibers, PE, dyed cotton and PP [11]. Magni et al. observed that plastic films accounted for 51% of microplastics in Italian sludge samples and acrylonitrile–butadiene copolymers were the main polymer fraction, followed by PP and polyester [16]. Additionally, it was found that polyurethane (PU) accounted for 28% of microplastic polymers in sludge, followed by polytetrafluoroethylene (PTFE) (14%), PE (12%) and PET (10%) [31].

The sludge treatment process often leads to the aging and fragmentation of micro(nano)plastics, as a result, the sizes of micro(nano)plastics in sludge are usually less than 500 μm [34,35,36]. Li et al. reported that the sizes of 75% of micro(nano)plastics in the sludge from a WWTP in China were between 20 and 100 μm [36]. Similarly, Li et al. found that micro(nano)plastics in sludge of a WWTP predominantly fell within the size of 10 to 500 μm, accounting for 98% of the total [31]. Liu et al. observed that all micro(nano)plastics particles were less than 1000 μm and over 70% of those ranged from 100 to 500 μm [15]. In contrast, other research showed that most micro(nano)plastics in sludge exceed 500 μm, and the size is mainly within the range of 500 to 5000 μm [5].

The micro(nano)plastics quantity in the sludge exhibits typical spatial-temporal differentiation. For example, it was reported that micro(nano)plastic concentrations in sludge from WWTPs in China was much higher than those in Europe and North America [14]. In China, micro(nano)plastic concentrations in sludge from eastern coastal regions was greater than that from western regions [32]. Furthermore, sludge from domestic WWTPs contained more micro(nano)plastics than those of industrial WWTPs [32]. Meanwhile, micro(nano)plastics content in sludge in summer was higher than that in winter [36]. In addition, Chaudhary et al. suggested that monsoon cycles also might influence micro(nano)plastics content in sludge and that this could be sorted in the following order: monsoon period > post-monsoon > pre-monsoon [37].

**Table 1:** Characteristics of micro(nano)plastics in sludge.

Location	Micro(nano)plastic Concentrations in WWTP Influent	Micro(nano)plastic Concentrations in WWTP Effluent	Sludge	Type of Micro(nano)plastics	Size of Micro(nano)plastics	Shape of Micro(nano)plastics	Micro(nano)plastics Quantity in Sludge	Reference
China	-	-	Activated sludge	PVC, PA, PP, PS, acrylates	20–100 μm (75%)	Fragments (most)	1710.67–6471.59 particles/g	[36]
China	2530–18,240 particles/L	650–1700 particles/L	Dewatered sludge	PU (28%), PTFE (14%), PE (12%), PET (10%)	<500 μm (98%)	Fibers, fragments, long fragments	38.6–104.5 particles/g	[31]
Japan	-	-	Dewatered sludge	-	100–500 μm (more than 70%)	-	6.47 ± 1.49 particles/g	[15]
Japan	-	-	WAS	-	100–500 μm (more than 70%)	-	81 ± 48 particles/kg	[15]
China	75–630 pieces/L	1–10 pieces/L	Activated sludge	-	-	-	7–888 pieces/g	[32]
China	-	1.6 ± 0.9 items/L	Dewatered sludge	-	<500 μm (78.3%)	-	6.4 ± 0.8 items/g	[35]
China	-	2.9 ± 0.8 items/L	Dewatered sludge	-	<500 μm (85.4%)	-	11.3 ± 2.3 items/g	[35]
Thailand	31.2 particles/L	14 particles/L	Dewatered sludge	-	50–1000 μm (most)	Fibers (20.4%), fragments (78.3%)	228.1 particles/g	[33]
China	0.70–8.72 items/L	0.07–0.78 items/L	-	PP, PE, PET, polyacrylonitrile (PAN)	500–5000 μm (most)	Fibers, fragments, films	234.7 ± 47.7–6908.3 ± 330.2 items/kg	[5]
Spain	-	12.8 ± 6.3 particles/L	Mixed sludge	Polyester fibers, acrylic fibers, PE, dyed cotton, PP	-	Fibers (62%)	183 ± 84 particles/g	[11]
Spain	-	12.8 ± 6.3 particles/L	Heat-dried sludge	Polyester fibers, acrylic fibers, PE, dyed cotton, PP	-	Fibers (84%)	165 ± 37 particles/g	[11]
Italy	2.5 ± 0.3 particles/L	0.4 ± 0.1 particles/L	Activated sludge	Co-polymers of acrylonitrile butadiene (27%), PP (18%), polyesters (15%)	100–500 μm	Films (51%), fragments (34%), lines (15%)	113 ± 57 particles/g	[16]
China	-	-	Dewatered sludge	Polyolefin, acrylic fibers, PE, PA, alkyd resin, PS	-	Fibers (63%), shafts (15%), films (14%), flakes (7.3%), spheres (1.3%)	1.60–56.4 particles/g	[14]

### 2.2. Food Waste

**Figure 3:**
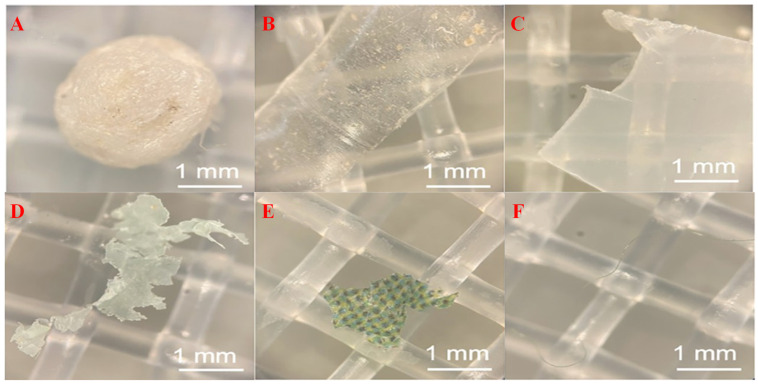
Micromorphology of microplastics in FW [41]: (**A**) PS fragment; (**B**) PET fragment; (**C**) PLA fragment; (**D**) PBAT film; (**E**) PE film; and (**F**) unidentified fiber.

Micro(nano)plastics in FW are an important pathway for plastic contaminants entering the AD system. Plastics found in FW mainly come from PE flexible packaging, PP rigid containers, PS foam boxes and PET beverage containers [38]. Table 2 provides detailed characteristics of micro(nano)plastics in FW. Plastic products are widely used in food packaging and processing, which become the main sources of micro(nano)plastics pollution in FW [39]. It was reported that about 38% of FW still retained intact packaging, and it was difficult to completely separate plastics from FW by mechanical or manual methods during the recycling process [12]. Furthermore, incomplete waste sorting may lead to the mixing of plenty of plastic waste into FW [40]. There are significant differences in the levels of micro(nano)plastics pollution in FW between different regions. In the Zhejiang province of China, micro(nano)plastic concentrations (mainly PE, PP, PET and PS) in household FW range from 5.78 × 10^3^ to 6.33 × 10^3^ N/kg (in urban, rural and town areas) [13]. In North America, the FW of Williston Transfer Station contains micro(nano)plastics at 3300 ± 1100 particles/kg total solid (TS) [41] (Figure 3). Micro(nano)plastics content in the biowaste of Austria ranges from 3% to 5.6% (dry weight), while that in the FW of Switzerland is only 0.12% (dry weight) [42,43]. It was reported that micro(nano)plastics accounted for 3–4% (wet basis) of FW from an FW treatment plant with a processing capacity of 200 tons per day in Hangzhou, China [44]. In an AD treatment plant of FW, the abundance of micro(nano)plastics (mainly PVC, PET, PP and PAN) reached 1.27 ± 0.43 × 10^3^ N/kg in the FW storage pit [45].

### 2.3. Livestock Manure

**Figure 4:**
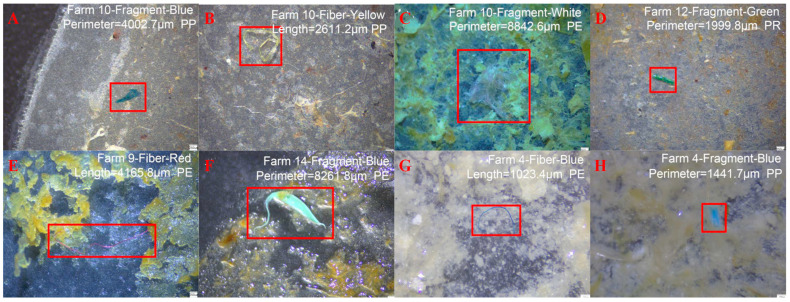
Micromorphology of microplastics in livestock manure [47]: (**A**–**E**) MPs detected in livestock manure from farmyards; (**F**) MPs detected in livestock manure from a layer breeding plant; (**G,H**) MPs detected in livestock manure from a pig plant.

Livestock manure is an important pathway of micro(nano)plastics entering the AD system (Figure 1). Micro(nano)plastics in livestock manure mainly originate from plastic components used in livestock management systems, including feed packaging, antibiotic containers and farm equipment [47]. These plastic products would degrade to micro(nano)plastics during daily use and enter the animal gastrointestinal system by feed, which could be detected in livestock manure (Figure 4). Current research reported that livestock manure contained a high level of micro(nano)plastics. Table 3 presents characteristics of micro(nano)plastics in livestock manure reported in previous studies. Wu et al. found that the microplastic content in pig manure and cow manure reached 9.02 × 10^2^ ± 1.29 × 10^3^ items/kg and 7.40 × 10^1^ ± 1.29 × 10^2^ items/kg, respectively, and suggested that polypropylene was the dominant plastic and accounted for 89% and 100% of microplastics in pig manure and cow manure [47]. Similarly, Lwanga et al. detected 129.8 ± 82.3 particles/g of microplastics in chicken manure, with the particle size of 0.1–1.0 mm [48]. Beriot et al. detected about 997 ± 971 particles/kg of microplastics in sheep manure from an agricultural system in Spain [49].

### 2.4. Other Organic Wastes

Municipal solid waste (MSW), green waste, stabilized organic waste and rural domestic waste are also rich in micro(nano)plastics, the characteristics of which are summarized in Table 4. Micro(nano)plastics in green waste were mainly composed of PP, LDPE and HDPE, and their abundance was affected by the seasons (5733 ± 850 particles/kg and 6433 ± 751 particles/kg in autumn and winter, respectively) [46]. It was reported that micro(nano)plastics abundance in stabilized organic waste output after mixed MSW processing reached 17,407 ± 1739 particles/kg in autumn and 15,400 ± 1217 particles/kg in winter [46]. MSW is an important source of micro(nano)plastics pollution in the urban environment. Edo et al. reported that micro(nano)plastics in MSW were mainly composed of PE, PS, polyester, PP, PVC and acrylic polymers, and their abundance ranged from 1 × 10^4^–3 × 10^4^ items/kg [50]. Similarly, Naeem et al. indicated that micro(nano)plastics in MSW consisted of PE, PP and PET and its concentration was 16,082 ± 632 particles/kg [51]. In contrast, micro(nano)plastics abundance in rural domestic waste was relatively low (about 2400 ± 358 items/kg), with the main components being PP and PE, mainly in the form of fibers and films [52].

## 3. Effect of Micro(nano)plastics on Anaerobic Digestion Performance

### 3.1. Polyvinyl Chloride (PVC) Micro(nano)plastics

PVC is widely produced around the world, and its annual yield accounted for about 12% of global plastics production [53]. Due to its low production cost, low price and good chemical stability, PVC is widely used in many fields such as agriculture, industry and daily life [54,55]. Only a small number of used PVC products are recycled, and most of them are discarded and enter organic waste streams through various pathways. Therefore, PVC inevitably enters the AD system along with organic waste and may affect the AD process performance, as shown in Table 5. It was found that the presence of PVC microplastics may significantly impact the hydrolysis and acidification of organics during AD, and PVC microplastic concentrations were the key factor governing their effects. Wei et al. found that 1.0 mm PVC microplastics with the concentration of 10 particles/g TS increased VFA production of AD of WAS by 4.9%, but a higher PVC microplastic concentration (20–60 particles/g TS) inhibited hydrolysis and acidification, leading to a reduction in VFA production by 6.9–16.8% [22]. In particular, PVC microplastics promoted sludge solubility, and the concentration of soluble chemical oxygen demand (SCOD) reached 3465 ± 25 mg/L in the digester with a PVC microplastic concentration of 60 particles/g TS and was 112.5% of the control [22]. Zhen et al. demonstrated that PVC microplastics at concentrations of 1, 5 and 10 mg/L promoted organic acidification and enhanced VFA concentrations by 7.82%, 13.89% and 13.31% during mesophilic (37 °C) AD of WAS [56].

The effects of PVC microplastics on the methane production of AD are highly dependent on their concentration in digesters. Multiple studies indicated that low-concentration PVC microplastics (e.g., ≤20 particles/g TS) slightly enhance the methane production of the AD of organic waste, whereas a high concentration of PVC microplastics exhibited an inhibition of methanogenesis. Wei et al. found that 1.0 mm PVC microplastics at a concentration of 10 particles/g TS increased the methane production of the AD of WAS by 5.9%, but PVC microplastics with high concentrations of 20–60 particles/g TS decreased the methane production by 9.4–24.2% [22]. This finding was in line with the report of Zang et al. that PVC microplastics (1.0 mm) with a concentration of 20 particles/g TS enhanced the methane production of the AD of WAS by 6.7%, and the increase in PVC microplastic (1.0 mm) concentration to 80 particles/g TS led to a great decline of methane production by 23.9% [57]. Similarly, Cheng et al. indicated that PVC microplastics (particle size of 166 μm) at a concentration of 40 mg/L slightly promoted the methane production of the AD of WAS (an increase of 1.64%), whereas PVC microplastics at a concentration of 200 mg/L significantly inhibited methane production (a decrease of 14.49%) [58]. Furthermore, Zhen et al. found that the temperature also affected the influence of PVC microplastics on the methane production of AD, and 150 μm PVC microplastics elevated methane production by 5.62–8.87% during mesophilic (37 °C) AD of WAS, but they decreased methane production by 3.30–19.99% under thermophilic AD (55 °C) [56]. In order to mitigate PVC microplastic inhibition on the AD of WAS, Tang et al. added cationic polyacrylamide into the digester and found that cationic polyacrylamide at 30 mg/g TS decreased the decrement of methane yield from 15.6% to 5.8% [59].

**Table 5:** Effects of PVC micro(nano)plastics on AD.

Micro(nano)plastics			AD Feedstock	Mode (Temperature)	Effects on AD	Reference
Type	Size	Concentration				
PVC	1.0 mm	10–60 particles/g TS	WAS	Batch (37 °C)	Microplastics at a concentration of 10 particles/g TS resulted in a 4.9% increase in VFA production and a 5.9% increase in methane production; microplastics at a concentration of 20–60 particles/g TS resulted in a 6.9–16.8% reduction in VFAs production and a 9.4–24.2% reduction in methane production.	[22]
PVC	1.0 mm	20, 80 particles/g TS	WAS	Batch (37 °C)	Microplastics at a concentration of 20 particles/g TS increased methane production by 6.7%, while microplastics at a concentration of 80 particles/g TS decreased methane production by 23.9%.	[57]
PVC	350 μm	30 mg/g TS	WAS	Batch (35 °C)	Microplastics inhibited VFA production by 15.2% and reduced methane production.	[59]
PVC	166 μm	40–200 mg/L	WAS	Batch (37 °C)	Microplastics at a concentration of 40 mg/L increased methane production by 1.64% while microplastics at a concentration of 200 mg/L decreased methane production by 14.49%.	[58]
PVC	150 μm	1–10 mg/L	WAS	Batch (37 °C)	Microplastics increased methane production by 5.62–8.87%.	[56]
PVC	150 μm	1–10 mg/L	WAS	Batch (55 °C)	Microplastics decreased methane production by 13.30–19.99%.	[56]

### 3.2. Polystyrene (PS) Micro(nano)plastics

PS is often found in various organic wastes and can be easily shredded by a slight mechanical force, after which it becomes PS micro(nano)plastics due to the loose structure of PS packaging [60]. The effects of PS micro(nano)plastics on AD performance are summarized in Table 6. Wang et al. found that the inhibition of PS microplastics on acidification during the AD of wastewater became stronger as the particle size of PS microplastics increased, and 150 μm PS microplastics exhibited the strongest inhibition (VFA production reduced by 38.3%) [61]. Qiao et al. proposed that PS microplastic addition did not alter the metabolic pathway responsible for VFA generation during the AD of FW, and a low concentration (10–20 mg/L) of PS microplastics increased VFA production, but a high concentration (50–200 mg/L) inhibited acidification [62]. Meanwhile, Wang et al. observed that 1 μm PS microplastics had a slight inhibitory effect on acidification during AD of WAS, 10 μm PS microplastics had almost no effect, but 50 nm PS nanoplastics repressed the acidification process [63]. Zhang et al. reported that PS micro(nano)plastics addition (≥0.25 g/L) significantly inhibited acidification during AD of synthetic wastewater and led to a decrease in the glucose degradation rate by 3.81–5.5% [64]. Li et al. demonstrated that the addition of PS microplastics inhibited VFA metabolism during the AD of FW, and PS microplastics with a smaller particle size exhibited stronger inhibition [40].

Current research also showed that PS micro(nano)plastics had significant effects on the methane production of the AD of organic waste. Wang et al. demonstrated that the inhibition of PS microplastics on methane production of the granular sludge AD system became more intense as the particle size increased and found that methane production reduced by 6.7–16.2% when the particle size of PS microplastics increased from 0.5 μm to 150 μm [61]. Conversely, Wang et al. found that 50 nm PS nanoplastics reduced the methane production of the AD of WAS by 15.5%, and 1 μm and 10 μm PS microplastics exhibited negligible effects on methane production and suggested that smaller PS nanoplastic particles exhibited stronger inhibition [63]. Qiao et al. investigated the effects of PS microplastics with the size of 100 μm on the AD of FW and found that PS microplastics with a concentration of 25 mg/L increased methane production by 4.72%, but methane production decreased by 10.13–17.18% when PS microplastic concentrations rose up to 50–200 mg/L [62]. Similarly, Zhang et al. found that PS micro(nano)plastics (particle sizes of 5 μm and 80 nm) at a low concentration (≤0.2 g/L) did not significantly affect the methane production of the AD of wastewater, but methane production declined 17.9–19.3% as PS micro(nano)plastic concentrations increased to 0.25 g/L [64]. Meanwhile, they found that small-size (80 nm) PS nanoplastic particles exhibited stronger inhibition to methane production than large-size PS microplastic particles [64]. Wei et al. observed that the exposure to 50 nm PS nanoplastics with concentrations of 20–50 mg/L for 86 days significantly reduced the methane production of the AD of wastewater by 19.0–28.6% [65]. Similarly, Li et al. reported that PS microplastics with a concentration of 20–200 particles/g TS reduced the methane production of the AD of FW by 1.46–33.08% [40]. Moreover, Zhao et al. indicated that the inhibition of aminated PS nanoplastics to methane production was significantly stronger than that of normal PS nanoplastics [66]. Current research showed that the effects of PS micro(nano)plastics on AD were closely related to particle size and the concentration of PS micro(nano)plastics. Generally, the higher the concentration of PS micro(nano)plastics, the greater the inhibition to methane production. At a low concentration (e.g., ≤25 mg/L), PS micro(nano)plastics (particle size of 100 μm) may promote methanogenesis by stimulating extracellular polymer secretion. However, when the concentration of PS micro(nano)plastics exceeds the threshold (e.g., 50 mg/L), a large amount of reactive oxygen species (ROS) induced by PS micro(nano)plastics may greatly inhibit methane production. When the concentration of PS micro(nano)plastics reaches the inhibition level in an anaerobic system, the smaller the particle sizes of PS micro(nano)plastics, the greater the inhibitory effects on methane production.

### 3.3. Polyethylene (PE) Micro(nano)plastics

PE accounts for about 30% of global plastic products that are widely used in plastic packaging, containers and personal care products [4,67]. PE inevitably enters the AD system along with organic wastes, which may affect AD process performance. Table 7 presents the effects of PE micro(nano)plastics on AD reported in previous studies. It was found that PE micro(nano)plastics might impact hydrolysis and acidification of organics during AD. Shi et al. investigated effects of PE microplastics with different sizes on the AD of WAS and found that 180 μm and 1 mm PE microplastics greatly enhanced VFA concentrations (1.7 and 7.1 folds compared to the control) and altered VFA distribution (180 μm PE microplastics increased butyrate production, and 1 mm PE microplastics promoted valerate production) [25]. Zhang et al. found that the VFA concentrations of the early AD of cattle manure with PE microplastics reached 157% of that of the control, and the peak of acetate concentration increased by 62.17%, and proposed that PE microplastics promoted organic hydrolysis and accelerated VFA production (especially acetate) [68]. In addition, Chen et al. found that thermal hydrolysis pretreatment could alleviate effects of PE microplastics hydrolysis and acidification during the AD of WAS [69].

Some studies showed that PE micro(nano)plastics had negative effects on methane production of AD. Shi et al. reported that 180 μm and 1 mm PE microplastics at a concentration of 200 particles/g TS decreased the methane production of the AD of WAS by 6.1% and 13.8%, respectively [25]. However, several studies reported the positive effects of PE micro(nano)plastics on AD. Zhang et al. found that PE microplastics (<400 μm) at a concentration of 1 g/L increased methane production by 8.4–41.2% during thermophilic AD of dairy waste [70]. Similarly, Zhang et al. indicated that the addition of 120 μm PE microplastics enhanced the methane production of the AD of cattle manure by 11.97% from 4972.59 ± 150.12 mL to 5568.05 ± 145.22 mL [68]. Akbay et al. investigated the effects of adding different sizes (50 μm and 150 μm) and concentrations (1–4 g/L) of PE microplastics on the biogas production of the AD of cosmetic industrial wastewater and found that 50 μm PE microplastics had no effect on biogas production, but 150 μm PE microplastics exhibited concentration-dependent effects: they increased biogas production by 12% at a concentration of 1 g/L; they had no effect at a concentration of 2 g/L; and they decreased biogas production by 7% at a concentration of 4 g/L [71]. Chen et al. explored the effects of thermal hydrolysis pretreatment on PE microplastics-induced stress in the AD of WAS and found that PE microplastics (at the concentration of 100 particles/g TS) inhibition to methane production was weakened by 31.4% from 12.1% to 8.3% by thermal hydrolysis pretreatment at 170 °C for 30 min [69]. PE microplastics with the same particle size but different concentrations often exhibit varying effects (promote, neutral or inhibit) on AD performance. The role of PE micro(nano)plastics in AD is closely related to particle size, concentration and substrate type.

### 3.4. Other Micro(nano)plastics

Except for PVC, PS and PE micro(nano)plastics mentioned above, other micro(nano)plastics such as PET, PP and PA micro(nano)plastics are also often found in anaerobic systems. Effects of these micro(nano)plastics on AD performance are summarized in Table 8. Most previous studies reported the negative effects of these micro(nano)plastics on AD. Wang et al. investigated the impact of PET microplastics with the concentration of 2.70 mg/g TS on the AD of sewage sludge with FW and found that 30 μm and 250 μm PET microplastics inhibited organic anaerobic degradation and reduced methane production by 21.63% and 15.87%, respectively [72]. Meanwhile, Wei et al. found that PET microplastics at a concentration of 60 particles/g TS suppressed bovine serum albumin, dextran, glucose and glutamate degradation by 20.3%, 13.2%, 11.6% and 82.2%, and decreased hydrogen production by 29.3% during the alkaline AD (pH 10.0) of WAS and demonstrated that PET microplastics inhibited hydrolysis, acidogenesis and hydrogen production [73]. Moreover, Wei et al. observed that four types of microplastics (PE, PET, PS and PP) had negligible effects on WAS dissolution during AD but significantly inhibited hydrolysis and acidification, resulting in a reduction in VFA production by 21.5 ± 0.1%. On the contrary, some reports indicate that these micro(nano)plastics may enhance AD performance [74]. Xu et al. found that 1 mm PA microplastics and PP microplastics with a concentration of 200 particles/g TS significantly improved sludge dissolution, hydrolysis, acidification and the methane production of the AD of WAS, as indicated by increased dissolved organic carbon, chemical oxygen demand and soluble protein concentrations and VFAs (the increment of 7.5% for PA microplastics and 32.6% for PP microplastics) and methane production (the increment of 11.7–35.5%) [75]. Xiao et al. also observed that the exposure to 150 μm PP microplastics at concentrations of 60–300 particles/g total suspended solids led to an increase (2.9–10.8%) of methane production [27]. Similarly, Chen et al. reported that PA6 microplastics at a concentration of 5–50 particles/g TS enhanced acidification (the peak of VFA concentration of the digester with PA6 microplastics at 10 particles/g TS increased by 23.5%) and increased methane production by 4.84–39.5% during the AD of WAS [24]. Current research indicated that micro(nano)plastics often exhibited inhibitory effects on the AD process and sometimes promoted methane production under a certain condition. Meanwhile, specific types of micro(nano)plastics, such as PA micro(nano)plastics and PP micro(nano)plastics, were often found to enhance methane production during AD.

## 4. Mechanisms of Micro(nano)plastics Affecting Anaerobic Digestion

### 4.1. Effects of Micro(nano)plastics on Microbial Cells

**Figure 5:**
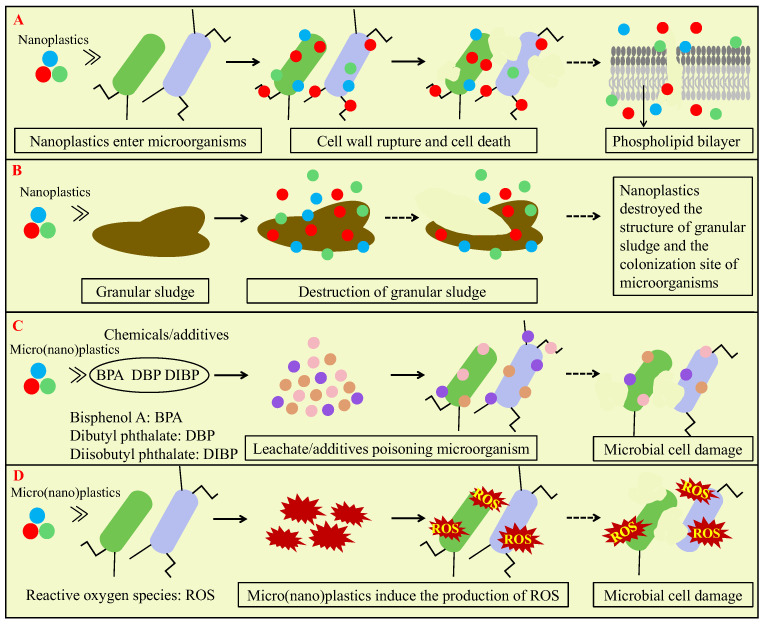
Mechanisms of micro(nano)plastics affecting AD via impacting microbial cells: disrupting cell membrane and entering cell interior (**A**); destruction of granular sludge (**B**); leachate/additives poisoning microorganisms (**C**); and inducing ROS (reactive oxygen species) production that damages microorganism cells (**D**).

The impact on microbial cells is an important pathway for micro(nano)plastics affecting AD, and the involved mechanisms mainly include disrupting the cell membrane, leachate/additives poisoning microorganisms and inducing ROS production that damages microorganism cells, as shown in Figure 5. In the AD system, the direct penetration of nanoplastics into microbial cells is a key mechanism. Nanoplastics attached to microbial cells may cause cell wall indentation and lead to cellular dysfunction with the gradual accumulation of nanoplastics inside the cell [1,76]. It was reported that micro(nano)plastics with the smaller size could enter the gaps between biopolymer chains more easily, penetrate microbial cell membranes and damage proteins and phospholipids [77]. For example, Fu et al. observed that 54.8 nm PS nanoplastics at a concentration of 0.25 g/L adhered to the cell membrane and induced nanopore formation due to the diapirism of nanoplastics in pure cultures of *Acinetobacter hydrogenigenes* [1]. Similarly, Zhang and Chen also confirmed that nanoplastics can enter cells and disrupt the integrity of the cell in the biofilm walls during wastewater and sewage sludge treatment [77]. Wei et al. demonstrated that exposure to 50 nm PS nanoplastics at a concentration of 20 mg/L or higher concentrations resulted in structural fragmentation of anaerobic granular sludge, and PS nanoplastics gradually accumulated on the surface of the granular sludge and subsequently migrated to deeper layers and poisoned the methanogens [65]. In addition, Dai et al. found that strong electrostatic interactions between positively charged PS nanoplastics and bacterial cell membranes can effectively promote their adsorption and endocytosis processes, leading to higher penetrability than neutral or negatively charged PS nanoplastics [78]. This direct membrane penetration process may cause physical damage to microorganisms and induce oxidative stress reactions.

When plastic products are produced, many additives are usually added to improve the performance of plastics. These additives or plastic monomers may leach out and impact AD performance, as shown in Figure 5C. These compounds may disrupt the cell membrane integrity of microorganisms or inhibit the activity of key antioxidant enzymes involved in AD. The toxicity of micro(nano)plastics to microorganisms is closely associated with the leached chemical additives. Wei et al. found that bisphenol A, as the main leaching additive of PVC microplastics, promoted methane production when the concentration of PVC microplastics was 10 particles/g TS but inhibited methane production when PVC microplastic concentrations were enhanced to 30–60 particles/g TS [22]. The reason might be that bisphenol A accelerated sludge dissolution by stimulating the decomposition of microbial cell walls and extracellular polymeric substances [22]. Wei et al. found that sodium dodecyl sulfate leached from PS nanoplastics (a particle size of 50 nm and a concentration of 50 mg/L) reduced methane production of AD of wastewater by about 6% and indicated that sodium dodecyl sulfate weakened the activity of key antioxidant enzymes of microorganisms, and superoxide dismutase and catalase activities decreased to 89.4 ± 2.3% and 85.6 ± 1.4% of the control group, which compromised the oxidative stress defense of the AD system against PS nanoplastics [65]. Meanwhile, Wang et al. found that diisobutyl phthalate and dibutyl phthalate leached from PET microplastics could inhibit the activity of hydrolytic acidification bacteria [72]. Chen et al. reported that caprolactam released from PA6 microplastics could strengthen methanogenesis by improving enzyme catalytic efficiency and increasing substrate affinity via binding to enzyme active sites [24]. Moreover, Jiang et al. indicated that hydrothermal pretreatment promoted the leaching of diisobutyl phthalate, dibutyl phthalate and bisphenol A from microplastics and inhibited methane production of AD of WAS [79]. However, Chen et al. demonstrated that thermal hydrolysis of PE microplastics increased the release of acetyltri-n-butyl citrate 8.6-fold and led to the significant increase in methane production (21.4%) [69]. In addition, several previous studies showed that micro(nano)plastics leachate had no significant effect on AD [23,63].

The microorganism cell apoptosis caused by ROS induced by micro(nano)plastics is also an important factor affecting AD performance [80]. The great surface area of micro(nano)plastics facilitates the attachment of reactive groups, which could stimulate the generation of free radicals and catalytic reactions with hydrogen peroxide and promote ROS formation. ROS induced by micro(nano)plastics mainly include hydrogen peroxide, superoxide and hydroxyl radicals, which could induce cellular oxidative stress [77]. In general, micro(nano)plastics with higher concentrations and smaller sizes induce the production of more ROS. Zhao et al. observed that PS microplastics at a low concentration (20–40 particles/g TS) did not significantly induce intracellular ROS production, while PS microplastics at a higher concentration (80–160 particles/g TS) obviously increased intracellular ROS levels in the AD system of WAS [81]. Wang et al. found that 150 μm PVC microplastics significantly increased ROS generation by 26.3 ± 4.0% in the anaerobic granular sludge wastewater treatment system, and the exposure to 75 μm and 150 μm PS microplastics promoted the release of lactate dehydrogenase to 145.2% and 188.4% of the control, which indicated a compromised cell membrane integrity [61]. Wang et al. observed that 1 μm and 10 μm PS microplastics had few effects on oxidative stress and cell survival in sludge flocs, but 50 nm PS nanoplastics significantly increased ROS levels in the AD system of WAS and reduced cell survival to 85.95 ± 0.27% of the control and suggested that nanoplastics with smaller particle size penetrated the extracellular polymeric substances more easily and induced stronger oxidative stress [63]. Similarly, Wei et al. indicated that 50 nm PS nanoplastics (at a concentration of 50 mg/L) increased ROS levels by 34.3 ± 0.1% during AD of wastewater, accompanied by a corresponding increase in lactate dehydrogenase release during short-term exposure to PS nanoplastics [65]. Li et al. also found that exposure to microplastics significantly increased the release of ROS and lactate dehydrogenase and suggested that microplastics inducing ROS generation might disrupt cell membrane structure and reduce cell viability [40]. Chen et al. found that the low-concentration bisphenol A leached from 30 particles/g TS polycarbonate microplastics reduced ROS production within cells, which enhanced enzyme activity, improved cell activity and increased the abundance of methanogens, thereby promoting methane production from the AD of WAS [82]. Conversely, a higher concentration of bisphenol A leached from 200 particles/g TS of polycarbonate microplastics stimulated ROS production, inhibited microbial cell activity and even induced cell apoptosis [82]. Huang et al. found that PE micro(nano)plastics with particle sizes of 100 μm and 100 nm induced excessive ROS production in microbial cells in the activated sludge system, which damaged cell membranes and decreased cell viability [83]. Meanwhile, Wei et al. found that PE microplastics at low concentrations (10–60 particles/g TS) did not lead to an obvious increase in ROS but that at high concentrations (100–200 particles/g TS) induced the significant increase in ROS production and the decrease in cellular activity by 7.6–15.4% and indicated that ROS production depended on microplastic concentrations [23].

### 4.2. Effects of Micro(nano)plastics on Key Enzymes

**Figure 6:**
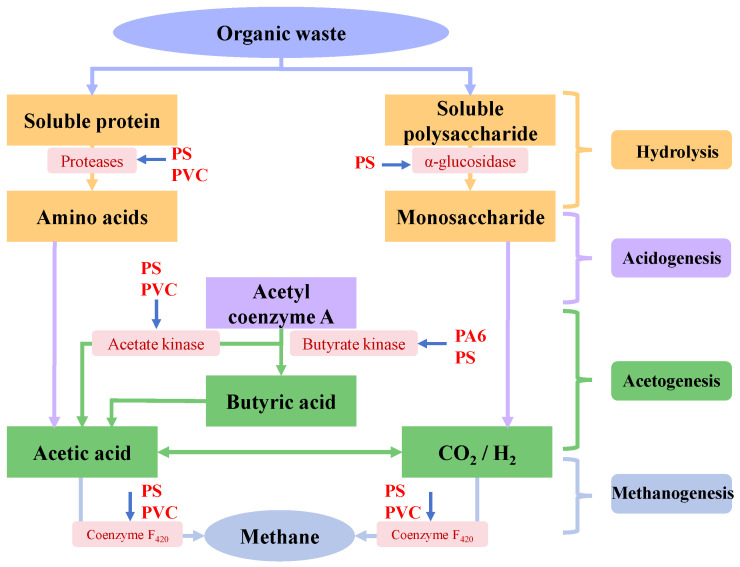
Effect of micro(nano)plastics on key enzymes involved in AD.

AD is a complex microbial process synergistically driven by multiple enzymes. The impact on key enzyme activities is an important pathway for micro(nano)plastics affecting AD, as shown in Figure 6. Among these enzymes, α-glucosidase and cellulase are indispensable for hydrolyzing polysaccharides, and proteases are necessary to degrade proteins. Acetate kinase plays an essential role in acetate production, and coenzyme F_420_ is critical in methanogenesis. Acetyl coenzyme A can be converted to acetate and butyrate by acetate kinase and butyrate kinase [22,24,40]. Generally, micro(nano)plastics with smaller sizes and higher concentrations exhibited stronger inhibition on key enzyme activity during AD. Wei et al. observed that PVC microplastics significantly inhibited enzyme activities of microorganisms in the AD system of WAS, especially PVC microplastics at a concentration of 60 particles/g TS, and led to a reduction in protease, acetate kinase and coenzyme F_420_ activities to 87.1 ± 0.2%, 87.2 ± 0.2%, and 79.3 ± 0.2% of the control, respectively [22]. Conversely, Wang et al. found that 50 nm PS nanoplastics enhanced the activities of protease, cellulase and coenzyme F_420_ during the AD of WAS but decreased the activity of acetate kinase from 632.5 ± 85.1 nmol/min/mL to 496.7 ± 40.6 nmol/min/mL [63]. Interestingly, Wang et al. found that adding 1 μm and 10 μm PS microplastics enhanced the activity of proteases and cellulases, which might portend the improvement of sludge hydrolysis [63]. However, the experiment result did not support this conclusion. The reason might be that the hydrolysis efficiency of sludge not only depended on enzyme activity but was also closely related to the distribution of active sites on the enzyme surface [22,24]. Li et al. indicated that PS microplastics inhibited the activities of multiple key enzymes in the anaerobic system of FW, and especially the inhibition rates of 1 μm PS microplastics on α-glucosidase, protease, acetate kinase and coenzyme F_420_ reached 79.10–98.08%, 78.00–98.08%, 81.95–95.60% and 82.24–96.21%, respectively [40]. These enzymes play an important role in major biochemical processes during AD, such as hydrolysis, acetylation and methanogenesis. Therefore, the exposure to PS micro(nano)plastics would impact metabolic functions of anaerobic microorganisms involved in these processes [40]. Zhao et al. found that low-concentration PS microplastics increased the activities of protease, α-glucosidase and coenzyme F_420_ during the AD of WAS, promoting the growth of acid-hydrolyzing bacteria, while high-concentration PS microplastics inhibited the activities of almost all enzymes and reduced the relative abundance of acetotrophic *Methanothrix*, leading to the decrease in methane production [81]. Meanwhile, Chen et al. also found that 5–50 particles/g TS of PA6 microplastics significantly affected key enzyme activities during the AD of WAS, and protease, butyrate kinase and coenzyme F_420_ activities exhibited parabolic changes with the increased PA6 microplastic concentrations in digesters [24]. They indicated that caprolactam leached from PA6 microplastics could bind with enzyme molecules and altered active sites of enzymes, which enhanced the affinity for substrates and catalytic activities of enzymes under the impact of low-concentration PA6 microplastics, while excess caprolactam occupied active sites of these enzymes under the impact of high-concentration PA6 microplastics (20 or 50 particles/g TS), thereby weakening the positive effects of PA6 microplastics on AD [24].

### 4.3. Effects of Micro(nano)plastics on Microbial Community

Anaerobic microorganisms play a decisive role in AD, and whether their ecological functions are exuberant is the key governing process performance. The ecological function of the anaerobic microorganism (especially methanogens) community is a significant point of penetration to explore the mechanisms of micro(nano)plastics affecting AD. It was reported that micro(nano)plastics might inhibit the growth and metabolism of multiple anaerobic microorganisms and lead to a decrease in their abundance. Wei et al. demonstrated that PVC microplastics significantly reduced the abundance of *Rhodobacter* and *Methanosaeta*, and the microbial community shifted toward the unfavorable conditions for hydrolysis, acidogenesis and methanogenesis [22]. Meanwhile, Wang et al. found that relative abundances of *Candidatus*_*Caldatribacterium*, *Methanobacterium* and *Methanosaeta* decreased by 114.8–39.7%, 15.1–40.4% and 60.2–74.4% when the particle size of PS microplastics in the anaerobic granular sludge wastewater treatment system was enhanced from 0.5 μm to 150 μm and indicated that the populations of key acidogens and methanogens were reduced, especially in the digesters affected by the larger micron-sized PS microplastics [61]. Wei et al. also reported that PS nanoplastics at concentrations of 20–50 mg/L reduced abundances of hydrolyzing and acidifying bacteria (typical for *Longilinea* and *Paludibacter*) in the AD system of wastewater, and, meanwhile, the abundance of methanogenic genera *Methanosaeta* and *Methanobacterium* decreased by 33.4–42.9% and 17.6–36.3%, respectively [65]. This result was in agreement with the findings of Li et al. that PS microplastics inhibited the growth of anaerobic microorganisms associated with methanogenesis, including *Synergistetes*, *Proteiniphilum*, *Methanosarcina* and *Methanothrix* [40]. Wei et al. proposed that long-term exposure to PE microplastics at a concentration of 200 particles/g TS reduced abundances of *Rhodobacter*, *Proteiniclasticum*, *Proteiniborus* and *Fonticella* by 15.2%, 24.1%, 28.2% and 19.4% during the AD of WAS [23]. Meanwhile, they found that the addition of PE microplastics with 200 particles/g TS reduced the total amount of methanogens but did not alter the aceticlastic methanogenic pathway dominated by *Methanosaeta* [23]. Furthermore, it was reported that the leaching of diisobutyl phthalate and dibutyl phthalate from PET microplastics led to a reduction in the abundances of key hydrolytic bacteria (*Bacteroides*vadin*HA17*) and acidogenic bacteria (*Clostridium* and *Sphaerochaeta*) [72].

Micro(nano)plastics were also proved to be beneficial to the growth of some specific anaerobic microorganisms. Fu et al. observed that microorganisms in the pure AD system exhibited differentiated tolerance to PS nanoplastics and the relative abundance of *Clostridiaceae*, *Geobacteraceae*, *Dethiosulfovibrionaceae* and *Desulfobulbaceae* was enhanced but the activities of *Cloacamonaceae*, *Porphyromonadaceae*, *Anaerolinaceae* and *Gracilibacteraceae* were inhibited [1]. Similarly, Wang et al. found that the addition of 50 nm PS nanoplastics to the anaerobic system of WAS resulted in a great decrease (36.6%) in the relative abundance of *Sulfurovum* but stimulated the growth of *Mariniphaga* and *Brevefilum* (relative abundance increased by 97.1% and 26.1%, respectively) [63]. Moreover, Xiao et al. suggested that PE microplastics not only enhanced the relative abundance of hydrolytic-acidifying bacteria (typical for *Syntrophobacter*, *Syntrophorhabdus* and *Syntrophomonas*) but also induced the significant growth of methanogens (typical for *Methanobacterium* and *Methanosaeta*) [27]. These previous studies confirmed the significant differences in responses of microbial communities to multiple micro(nano)plastics in various AD systems. The mechanisms of long-term exposure to micro(nano)plastics affecting the microbial community should be further investigated.

### 4.4. Effect of Micro(nano)plastics on Antibiotic Resistance Genes

**Figure 7:**
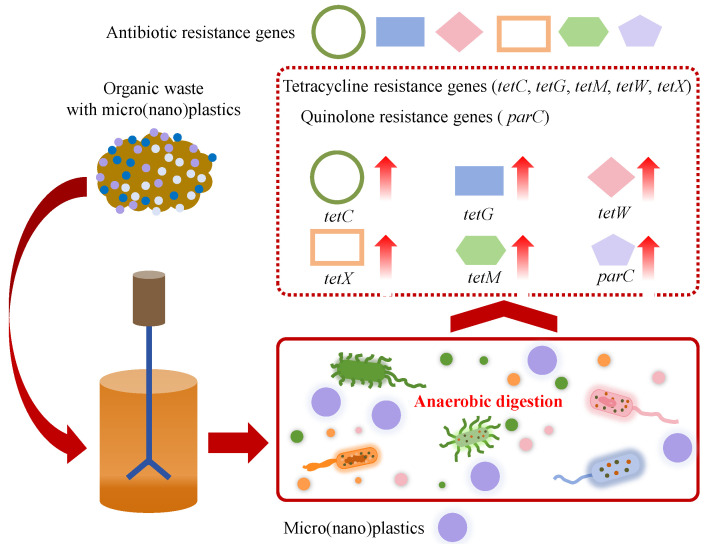
Effects of micro(nano)plastics on ARGs in the anaerobic system.

Some organic wastes (typical for sludge and livestock manure) contain significant amounts of ARGs such as tetracycline resistance genes (*tetA*, *tetB*, *tetP*, *tetC*, *tetE*, *tetG*, *tetM*, *tetO*, *tetQ*, *tetT*, *tetW*, *tetX*, etc.), macrolide resistance genes (*ermB*, *ermC*, etc.), sulfonamide resistance genes (*sul1*, *sul2*, etc.), quinolone resistance genes (*qepA*, *oqxB*, *parC*, etc.), beta-lactam resistance genes (*bla_OXA_*, *bla_TEM_*, etc.) and integron integrase genes (*intI1*, *intI2*, etc.) [27,70,84]. During AD, ARGs carried by organic wastes enter the AD system along with micro(nano)plastics. The presence of micro(nano)plastics influences the abundance of ARGs in AD, as shown in Figure 7. Shi et al. found that ARG abundance in the AD system of WAS with the addition of 180 μm PE microplastics reached 7.6 × 10^8^ (*sul2*), 1.3 × 10^7^ (*bla_OXA_*), 2.0 × 10^8^ (*tetW*), 6.0 × 10^8^ (*tetO*) and 1.0 × 10^7^ (*tetQ*) copies/g sludge, and those of the digester, with the addition of 1 mm PE microplastics, were 9.6 × 10^8^ (*sul2*), 2.2 × 10^7^ (*bla_OXA_*), 2.3 × 10^8^ (*tetW*), 8.8 × 10^8^ (*tetO*) and 1.4 × 10^7^ (*tetQ*) copies/g sludge, indicating that PE microplastics induced the enrichment of ARGs [25]. In detail, compared to the control without microplastic addition, the addition of 180 μm PE microplastics increased total ARG abundance 1.2–3.0-fold, while the addition of 1 mm PE microplastics induced a greater increase in ARG abundance (1.5–4.0-fold) [25]. Similarly, Zhang et al. reported that PE microplastics significantly increased the fold changes of *tetC*, *tetG*, and *tetW* in the AD of dairy wastes from 0.71 to 12.63 to 1.15–28.62 [70]. Xiao et al. found that PP microplastics induced the increase in the abundance of *tetX* (13.1–73.6%), *tetM* (1.5-fold) and *parC* (35.5–222.7%) in the AD system and also induced the enrichment of mobile genetic elements such as *ISCR1* and *intI1* [27]. Li and Yuan observed that polylactic acid (PLA) microplastics, PE microplastics and PET microplastics with an approximate particle size of 100 μm increased the abundance of ARGs in the AD of sludge by 14.15%, 29.90% and 18.64%, and meanwhile induced the enrichment of mobile genetic elements [26]. Moreover, Luo et al. reported that microplastics enhanced the prevalence of ARGs in anaerobic sludge digestion by enriching antibiotic-resistant bacteria [85]. The abundances of ARGs demonstrated a dosage-dependent relationship within the range of 10 to 80 particles/g TS of microplastics, resulting in an increase from 4.5 to 27.9% compared to the control [85]. Wang et al. found that 40–48 μm PE microplastics at concentrations of 50–200 particles/g TS promoted the proliferation of ARGs and mobile genetic elements during thermophilic AD of sewage sludge and PE microplastics; ARGs and mobile genetic elements exhibited strong positive correlations [86]. The enrichment of some functional genus occurred under PE microplastic stress, which might be the potential host of ARGs and mobile genetic elements [86]. Furthermore, PET microplastic fibers at the concentration of 170 items/g TS were reported to significantly increase the abundance of extracellular ARGs during the AD of sewage sludge, and the absolute and relative abundances of extracellular ARGs were 1.70- and 2.15-fold higher than those in the control [87]. In addition, it was reported that some plastic additives (typical for plasticizers), such as dimethyl phthalate, could promote the spread of ARGs during the AD of sludge [88]. Meanwhile, the aging of micro(nano)plastics also influences the enrichment of ARGs in the AD system. Haffiez et al. found that the relative abundance of ARGs in digesters exposed to aged PS nanoplastics was higher than those exposed to non-aged PS nanoplastics [84]. Most previous studies reported that the presence of micro(nano)plastics in the AD system would enrich ARGs and increase the spread risk and the potential health threat. Interestingly, several studies proposed that micro(nano)plastics might reduce the abundance of ARGs during AD. Xu et al. found that the exposure to PA microplastics, PE microplastics and PP microplastics resulted in a decrease in the total abundance of ARGs by 5.6–24.6%, accompanied by an increase in methane production by 11.7–35.5% in the AD of WAS [75]. The interaction mechanism between micro(nano)plastics and ARGs during the AD of organics should be further explored.

## 5. Coupling Effects of Micro(nano)plastics with Other Pollutants on Anaerobic Digestion

Micro(nano)plastics, heavy metals, antibiotics and other emerging contaminants (perfluorooctanoic acid, perfluorooctane sulphonate, persistent organic pollutants, endocrine-disrupting chemicals, etc.) are often found together in organic wastes and AD systems [89,90]. Micro(nano)plastics with a large specific surface area would adsorb heavy metals, ARGs and other emerging contaminants and act as carriers of these pollutants, which exhibit coupling effects on the AD of organics [79]. It was reported that micro(nano)plastics adsorbing antibiotics and heavy metals enhanced the inhibitory effect of micro(nano)plastics on microorganisms [91,92,93]. Kong and Shi investigated the effects of co-exposure to different microplastics (PE, PS, PVC and PET) and sulfamethoxazole on AD performance and microbial communities in the anaerobic system of synthetic wastewater and found that co-exposure of microplastics with sulfamethoxazole slightly decreased the chemical oxygen demand removal rate and methane production and decreased the relative abundance of *Euryarchaeota*, which ranged from 1.88% to 4.63% [94]. Similarly, Xiang et al. found that co-exposure to PA nanoplastics and ofloxacin resulted in an increase in the abundance of five ARGs by 4.4–359.1% and, compared to exposure to ofloxacin alone and ofloxacin combined with PA nanoplastics, promoted the formation of biofilm and enhanced adhesion and communication among microorganisms during the AD of sludge [95]. Xiang et al. indicated that the coexistence of PA microplastics enabled partial adsorption of ofloxacin and greatly mitigated the inhibition of low-concentration ofloxacin (20 mg/L) to the AD of sludge, leading to an obvious increase (21.0%) in methane production compared to the existence of ofloxacin alone [96]. Nevertheless, Xiang et al. also suggested that methanogens were completely inhibited by high-concentration ofloxacin (200 mg/L), and the addition of PA microplastics could not alleviate the fierce ofloxacin inhibition of methanogenesis [96].

When micro(nano)plastics and heavy metals coexist in complex environmental systems, micro(nano)plastics may adsorb and facilitate the migration of heavy metals and exhibit co-toxicity [97]. Qi et al. confirmed that the particular surface structure of microplastics promoted the formation of biofilm, which enhanced the adsorption of Pb(II) onto microplastics and enhanced the combined toxicity of Pb(II) and microplastics [98]. Liu et al. investigated the combined effects of microplastics and Cd on the AD of sewage sludge and found that the Cd element at a concentration of 5 mg Cd/g TS decreased methane production to 80 ± 5 mL/g VS (only 58.8% of the control) and the addition of PVC microplastics at concentrations of 1, 10 and 30 particles/g TS increased methane production to 66.9%, 86.8% and 89.7% of the control [99]. They indicated that PVC microplastics adsorbed the Cd element and reduced Cd bioavailability in sewage sludge by anaerobes and therefore significantly alleviated Cd toxicity to methanogens [99]. So far there is still a lack of comprehensive and systematic understanding concerning coupling effects of micro(nano)plastics with heavy metals, antibiotics and other emerging contaminants (perfluorooctanoic acid, perfluorooctane sulphonate, persistent organic pollutants, endocrine-disrupting chemicals, etc.) on AD, which should therefore be further investigated in the future.

## 6. Conclusions

Common organic waste, such as sludge, FW, livestock manure, green waste (yard waste) and municipal solid waste, is rich in multiple micro(nano)plastics and is the main pathway of micro(nano)plastics entering the anaerobic system. Micro(nano)plastics have significant impacts on AD processes, which have become an important research hotspot in the fields of environmental science and biotechnology. Their effects on AD performance highly depend on their physical and chemical properties and the operating conditions of the anaerobic system. Micro(nano)plastics have many types (PVC, PS, PE, PP, PET, etc.) and diverse physical (size, shape, pore, micromorphology, structural strength, etc.) and chemical (chemical composition, additive, leaching characteristics, etc.) properties, and thus exhibit different effects on AD performance. Most current studies suggested that micro(nano)plastics could reduce organic hydrolysis rates, inhibit VFA degradation and decrease methane production. The potential mechanisms of micro(nano)plastics affecting AD focus on micro(nano)plastic particles and their leachates (inducing ROS production) penetrating or damaging cellular structures and causing microorganism death, inhibiting the activities of key enzymes (coenzyme F_420_, acetyl coenzyme A, etc.) involved in methanogenesis, altering microorganism community structures and aggravating the enrichment of ARGs. Crucially, micro(nano)plastics adsorbing other contaminants (antibiotics, heavy metals, etc.) may lead to complex coupling impacts on anaerobic systems and increase environmental risk. In the future, more in-depth explorations in regard to the mechanisms of micro(nano)plastics affecting organics metabolic pathways and the expression of specific functional genes of microorganisms, fate and transformation of micro(nano)plastics along waste streams, the interaction between micro(nano)plastics and other emerging contaminants (perfluorooctanoic acid, perfluorooctane sulphonate, persistent organic pollutants, endocrine disrupting chemicals, etc.) and their coupling effects on the anaerobic system, accurate detection and quantification methods for micro(nano)plastics, as well as effective strategies for eliminating negative impacts of micro(nano)plastics on AD, should be carried out. In addition, micro(nano)plastics in organic wastes not only affect the AD process but also may enter agricultural soil along with the digestate, which would impact the ecological environment.

## Figures and Tables

**Table 2:** Characteristics of micro(nano)plastics in FW.

FW	Type of Micro(nano)plastics	Size of Micro(nano)plastics	Shape of Micro(nano)plastics	Micro(nano)plastics Quantity in FW	Reference
Grocery waste	-	-	-	3 × 10^5^ particles/kg	[12]
Household FW	PE, PP, PET, PS	>1000 μm (50%)	Fibers, films	5.78–6.33 × 10^3^ N/kg	[13]
Restaurant FW	PE, PP, PET	>5000 μm (40.28%)	Fibers (86%)	11.78 ± 0.88 × 10^3^ N/kg	[13]
FW	PVC, PET, PAN, PP	<200 μm (71.8%)	Granule (49.5%), fibers (34.2%)	1.27 ± 0.43 × 10^3^ N/kg	[45]
FW (autumn)	Low-density polyethylene (LDPE), high-density polyethylene (HDPE), PS, PET, PP	<500 μm (73%)	Fragments, fibers, films, spheres	3783 ± 351 particles/kg	[46]
FW (winter)	LDPE, HDPE, PS, PET, PP	<500 μm (73%)	Fragments, fibers, films, spheres	4066 ± 658 particles/kg	[46]
Depackaged food scraps	-	-	-	3300 ± 1100 particles/kg TS	[41]

**Table 3:** Characteristics of micro(nano)plastics in livestock manure.

Livestock Manure	Type of Micro(nano)plastics	Size of Micro(nano)plastics	Shape of Micro(nano)plastics	Micro(nano)plastics Quantity in Livestock Manure	Reference
Pig manure	PP (89%)	-	Fragments, fibers	9.02 × 10^2^ ± 1.29 × 10^3^ items/kg	[47]
Cow manure	PP (100%)	-	Fragments, fibers	7.40 × 10^1^ ± 1.29 × 10^2^ items/kg	[47]
Pig manure	Polyester (PES), PP, PE, rayon	1000–5000 μm (63.2%)	Fibers (79.2%)	1250 ± 640 particles/kg	[17]
Sheep manure	-	-	-	997 ± 971 particles/kg	[49]
Chicken manure	-	100–1000 μm	-	129.8 ± 82.3 particles/g	[48]
Pig manure	PE, PP, PET	<500 μm (Most)	Fibers	2.22 ± 0.16 × 10^3^ N/kg	[13]
Cow manure	PE, PP, PET, PS	<500 μm	Fibers	1.89 ± 0.31 × 10^3^ N/kg	[13]

**Table 4:** Characteristics of micro(nano)plastics in other organic wastes.

Organic Waste	Type of Micro(nano)plastics	Size of Micro(nano)plastics	Shape of Micro(nano)plastics	Micro(nano)plastics Quantity in Organic Waste	Reference
Green waste (autumn)	PP (50%)	<500 μm (86%)	Films (55.2%)	5733 ± 850 particles/kg	[46]
Green waste (winter)	PP (50%)	<500 μm (86%)	Films (56.2%)	6433 ± 751 particles/kg	[46]
Stabilized organic waste (autumn)	PP (29%), LDPE (17%), HDPE (21%)	<500 μm (81.5%)	Fragments (about 50%)	17,407 ± 1739 particles/kg	[46]
Stabilized organic waste (winter)	PP (29%), LDPE (17%), HDPE (21%)	<500 μm (81.5%)	Fragments (about 50%)	15,400 ± 1217 particles/kg	[46]
Rural domestic waste	Polyester PP, PE	<1000 μm (most)	Fibers, films	2400 ± 358 items/kg	[52]
Municipal solid waste	PE, PS, polyester, PP, PVC, acrylic polymers	-	-	1 × 10^4^–3 × 10^4^ items/kg	[50]
Municipal solid waste	PE, PP, PET	<100 μm (most)	Fibers, fragments	16,082 ± 632 particles/kg	[51]

**Table 6:** Effects of PS micro(nano)plastics on AD.

Micro(nano)plastics			AD Feedstock	Mode (Temperature)	Effects on AD	Reference
Type	Size	Concentration				
PS	0.5, 1, 10, 50, 75, 150 μm	75 mg/L	Wastewater	Batch (35 °C)	Methane production decreased by 6.7–16.2%.	[61]
PS	100 μm	25–200 mg/L	FW	Batch (35 °C)	Microplastics with a concentration of 25 mg/L increased methane production by 4.72%. Microplastics with a concentration of 50–200 mg/L decreased methane production by 10.13–17.18%.	[62]
PS	50 nm, 1.0 μm, 10 μm	-	WAS	Batch (35 °C)	Both 1 μm and 10 μm PS microplastics did not significantly affect methane production. Whereas 50 nm PS nanoplastics decreased methane production by 15.5%.	[63]
PS	80 nm, 5 μm	0.05–0.25 g/L	Synthetic wastewater	Batch (35 °C)	PS micro(nano)plastics with a low concentration (≤0.2 g/L) had no effect on methane production, while PS micro(nano)plastics with a concentration of 0.25 g/L resulted in an obvious decrease in methane production (17.9–19.3%).	[64]
PS	54.8 nm	0.05–0.25 g/L	Sewage sludge	Batch (37 °C)	Methane production decreased by 4.7–14.4%.	[1]
PS	50 nm	10–50 mg/L	Wastewater	Continuous (35 °C)	The long-term exposure (86 days) of PS nanoplastics with concentrations of 20 and 50 mg/L led to a great reduction in methane production (19.0–28.6%).	[65]
PS	1 μm, 100 μm, 1 mm	20 and 200 particles/g TS	FW	Batch (37 °C)	Both 20 particles/g TS and 200 particles/g TS of microplastics resulted in a decrease in methane production by 1.46–18.11% and 9.14–33.08%, respectively.	[40]
Aminated PS	80 nm	60 mg/g TS	WAS	Batch (37 °C)	Aminated PS nanoplastics reduced the hydrolysis rate and methane production.	[66]

**Table 7:** Effect of PE micro(nano)plastics on AD.

Micro(nano)plastics			AD Feedstock	Mode (Temperature)	Effects on AD	Reference
Type	Size	Concentration				
PE	40 μm	10–200 particles/g TS	WAS	Batch (37 °C)	PE microplastics at concentrations of 100–200 particles/g TS decreased methane production by 12.4–27.5%.	[23]
PE	40 μm	100 and 200 particles/g TS	WAS	Continuous (37 °C)	Average daily methane production was 28.8% lower than that of the control.	[23]
PE	180 μm, 1 mm	about 200 particles/g TS	WAS	Batch (37 °C)	Both 180 μm and 1 mm PE microplastics decreased methane production by 6.1% and 13.8%, respectively.	[25]
PE	< 400 μm	1 g/L	Dairy waste	Batch (55 or 65 °C)	PE microplastics increased methane production by 8.4% and 41.2% during thermophilic (55 and 65 °C, respectively) AD.	[70]
PE	50 μm, 150 μm	1–4 g/L	Cosmetic industry waste	Batch (36 °C)	PE microplastics with a concentration of 4 g/L reduced biogas production by about 7%. PE microplastics with a concentration of 1 g/L increased biogas production by about 12%.	[71]
PE	150 μm	10 and 100 particles/g TS	WAS	Batch (37 °C)	PE microplastics at a concentration of 100 particles/g TS led to a reduction in methane production of 12.1%. Thermal hydrolysis pretreatment greatly alleviated PE microplastics inhibition to methane production.	[69]

**Table 8:** Effect of other types of micro(nano)plastics on AD.

Micro(nano)plastics			AD Feedstock	Mode (Temperature)	Effects on AD	Reference
Type	Size	Concentration				
PET	30 μm, 250 μm	0.45–2.70 mg/g TS	Sewage sludge and FW	Batch (37 °C)	Both 30 μm and 250 μm PET microplastics at a concentration of 2.70 mg/g TS decreased methane production by 21.63% and 15.87%, respectively.	[72]
PET	25 μm	10–60 particles/g TS	WAS	Batch (37 °C)	PET microplastics at the concentration of 60 particles/g TS decreased hydrogen production by 29.3%.	[73]
PE, PET, PS, PP	-	24, 25, 7 and 5 particles/g TS	WAS	Batch (21 °C)	Microplastics significantly inhibited hydrolysis and acidification of organics.	[74]
PA, PP	1 mm	200 particles/g TS	WAS	Batch (35 °C)	Microplastics increased methane production by 11.7–35.5%.	[75]
PP	150 μm	60–300 particles/g TS	Granular sludge	Batch (37 °C)	Microplastics increased methane production by 2.9–10.8%.	[27]
PA6	0.5–1.0 mm	5–50 particles/g TS	WAS	Batch (37 °C)	Microplastics enhanced methane production by 12.9–39.5%.	[24]

## Data Availability

No new data were created or analyzed in this study. Data sharing is not applicable to this article.
